# World Health Organization meningioma grade II

**DOI:** 10.11604/pamj.2022.42.192.34663

**Published:** 2022-07-08

**Authors:** Rutuja Bhaskar Parkhi, Snehal Subrat Samal

**Affiliations:** 1 Department of Neurophysiotherapy, Ravi Nair Physiotherapy College, Datta Meghe Institute of Medical Sciences, Sawangi, Meghe, Wardha, Maharashtra, India

**Keywords:** Meningioma, brain tumor, neoplastic etiology

## Image in medicine

We are presenting to you a Magnetic Resonance Imaging (MRI) finding of a 65 years old female who presented to us with a complain of headache which was over the frontal region and on and off in nature. The patient also complains of imbalance during walking and tingling, numbness and weakness in the left upper and lower extremities. She gives a history of 2 episodes of seizures. Contrast magnetic resonance imaging findings revealed a well-defined extra-axial lesion measuring approximately 4.1*3.9cm (red arrow and green arrow) in the parafalcine region at the right frontoparietal lobes with mass effect (blue arrow). In contrast, the lesion shows avid peripheral enhancement with tiny non-enhancing central core features suggesting neoplastic etiology (yellow arrow), i.e., World Health Organization meningioma grade II. Meningiomas, classified as grade 2 by the World Health Organization, are aggressive tumors with a high recurrence rate that necessitates multiple surgical procedures and can significantly worsen a patient's neurological condition. The lesion is hyperintense and homo-dense near the coronal suture. She underwent surgical excision of the right frontoparietal parasagittal tumor and is under regular physiotherapeutic and medical management for the residual weakness.

**Figure 1:**
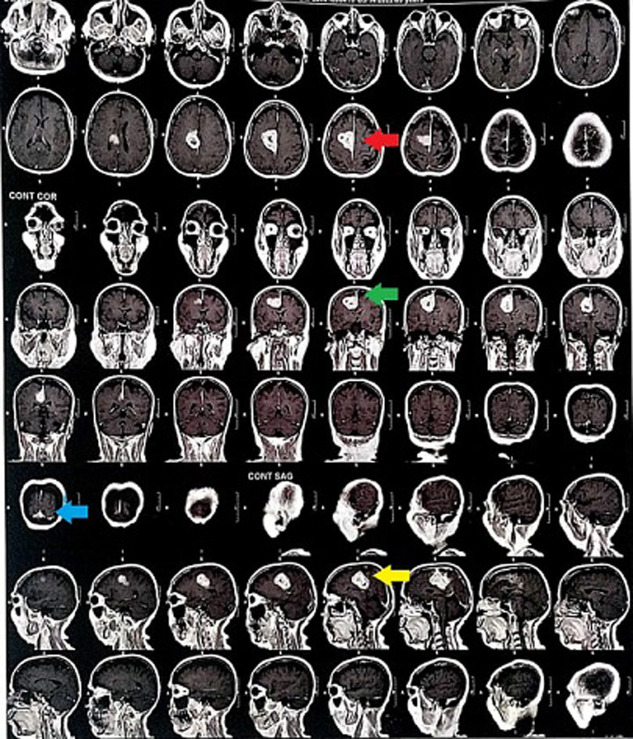
MRI findings of grade II meningioma; red and green arrows show extra-axial lesions measuring approximately 4.1*3.9cm; the blue arrow shows the mass effect; the yellow arrow shows neoplastic etiology

